# Impact of nanochelated fertilizers on enhancing growth, photosynthesis, antioxidant defense, and yield of wheat and oat under salinity stress

**DOI:** 10.3389/fpls.2026.1867951

**Published:** 2026-06-26

**Authors:** Anna Harutyunyan, Abhishek Singh, Hrant Khachatryan, Ashot Amirkhanyan, Hasmik Karapetyan, Anuj Saraswat, Karen Ghazaryan

**Affiliations:** 1 Applied Ecology and Environmental Research Laboratory, Research Institute of Biology, Faculty of Biology, Yerevan State University, Yerevan, Armenia; 2 Basic and Pathological Biochemistry Laboratory, Research Institute of Biology, Faculty of Biology, Yerevan State University, Yerevan, Armenia; 3 Department of Biology, College of Science, United Arab Emirates University, Al Ain, United Arab Emirates

**Keywords:** antioxidants enzymes, nano-chelated fertilizers, oxidative stress, photosynthesis, plant growth, salinity stress, wheat and oat

## Abstract

Salinity stress is a major constraint limiting crop productivity by inducing osmotic imbalance, ion toxicity, and oxidative damage. The present study evaluated the effectiveness of nanochelated fertilizers, including potassium-rich NPK (12–12–36; NPK_1_), balanced NPK (20–20–20; NPK_2_), and a micronutrient complex, on wheat and oat under varying salinity levels in a pot experiment, categorized as Non-saline (EC_e_=1.7 dS m^-1^), Slight-saline (EC_e_=2.9 dS m^-1^), Moderate-saline (EC_e_=6.2 dS m^-1^), and High-saline (EC_e_=12.5 dS m^-1^). Salinity significantly reduced growth and physiological traits. In wheat, shoot height decreased by 17.4% and biomass by 41.8% under high salinity, while in oats, reductions reached 23.5% and 41.0%, respectively. Photosynthetic rate (Pn) declined by 54.6% in wheat and 49.1% in oats, while chlorophyll content index (CCI) decreased by 23.0% and 51.9%, respectively. In contrast, oxidative stress markers increased substantially, with MDA rising 4.11-fold in wheat and 3.00-fold in oats, and proline increasing 1.80-fold and 3.37-fold, respectively. Application of nanochelated fertilizers mitigated these adverse effects across all salinity levels. According to morphological parameters, the more significant results were observed in wheat when applying a combination of NPK_1_ and micronutrient treatment, and in oats when applying NPK_1_ treatment. In particular, in wheat, the decrease in shoot height was 5.8%, in oats, 17.8%, in biomass, 25.0% (wheat) and 24.5% (oats). In the case of physiological parameters, according to Pn values, the combination of NPK_2_ and micronutrients treatment in the case of wheat and the NPK_2_ treatment in the case of oats had the most positive effect from nanochelated fertilizers, reducing the decline in Pn to 17.9% (wheat) and 10.0% (oats) under high salinity. The positive effect of nanochelated fertilizers on CCI values ​​was seen in the NPK_2_ treatment for both crops, with reductions limited to 12.5% ​​in wheat and 27.0% in oats. The combined treatment of NPK_2_ and micronutrients showed the most pronounced improvements in lipid peroxidation and enzymatic antioxidant activity. Lipid peroxidation stress marker MDA levels decreased by 1.29-fold (wheat) and 1.61-fold (oats) compared to high-saline controls. Antioxidant enzyme activities were significantly enhanced, with CAT increasing up to 1.27-fold (wheat) and 1.19-fold (oats), and SOD up to 1.26-fold and 1.21-fold, respectively. Proline amount also increased up to 1.13-fold (wheat) and 1.16-fold (oat). Yield-related traits declined under stress, with TKW decreasing by 46.7% in wheat and 17.1% in oats, but combination of NPK_1_ and micronutrients treatment in wheat, and micronutrients treatment in oats, reduction was limited to 23.3% ​​in wheat and 6.7% in oats, compared to the Non-saline Control. Nanochelated fertilizers enhanced salinity tolerance by improving growth, photosynthetic efficiency, antioxidant defense, and ion regulation, showing potential for sustainable crop production under saline conditions.

## 1 Introduction

In the early 21^st^ century, challenges such as severe water shortages, environmental pollution, and increasing soil and water salinity. The expanding human population, coupled with decreasing arable land, poses significant threats to agricultural sustainability (Shahbaz and Ashraf, 2013). The FAO recently released its first comprehensive worldwide survey of salt-affected soils in half a century (FAO, 2024). According to the report, a total of almost 1.4 billion hectares of land (slightly more than 10% of the world’s land surface) are already affected by salinization, and another one billion hectares have a ‘significant potential to be impacted’ as a result of the climate crisis and mismanagement by humans. The estimated area of salt-affected soils was about 1381 million ha (Mha), representing about 10.7% of the total global land area, which has a significant impact on agriculture globally (FAO, 2024). It also estimates that salinity affects 10% of irrigated cropland and about 10% of rainfed cropland, but these figures may be higher due to limited data availability. Trends in global aridity models show that under the current temperature rise trend, the area affected could reach 24%-32% of the land surface. Widespread aridification will be concentrated in developing countries. 70% of the salt-affected soils in the world are in 10 countries, e.g., Afghanistan, Australia, Argentina, China, Kazakhstan, the Russian Federation, the United States of America, Iran, Sudan and Uzbekistan (FAO, 2024). Saline soils are defined by an electrical conductivity (EC) of the saturation extract (EC_e_) above 4 ds/m^−1^ in the root zone at 25 °C, and an exchangeable sodium percentage (ESP) exceeding 15% (Munns, 2005; Jamil et al., 2011).

Salinity affects many stages of plant development, including seed germination, vegetative growth, and the reproductive process (Bano and Fatima, 2009; Azeem and Wenxuan, 2026). It induces negative alteration for morphological, physiological, and biochemical traits because of salinity ion toxicity, osmotic stress, nutrient stress deficiencies of essential nutrients (e.g., nitrogen, calcium, potassium, and phosphorus, but also iron and zinc, etc.) and oxidative stress, which all ultimately limit water uptake from the soil (Munns, 2002). A major effect of salinity is a decrease in phosphorus uptake, since phosphate tends to precipitate with calcium (Penn and Camberato, 2019). Also, hyperaccumulation of sodium in cell walls is a potential cause of osmotic stress, which can lead to plant cell death (Munns, 2002). In addition, high levels of extreme reactive oxygen species (ROS) (e.g., O_2_^•−^, ^1^O_2_, H_2_O_2_ and OH^•^) production induce more serious damage at the subcellular level because their accumulation in plant tissues is associated with salinity stress-induced osmotic and ionic stresses (Hasanuzzaman et al., 2020). An excessive production of ROS causes lipid peroxidation and the formation of malondialdehyde (MDA), which interferes with cellular metabolism and physiological functions (Hasanuzzaman et al., 2020). This leads to a detrimental impact on membrane structure and functions, including maintaining cellular integrity and transport of molecules, cell signaling, and compartmentalization of cellular processes (Munns and Tester, 2008). In addition to osmotic stress and ionic disturbance, salinity also produces a nutritional imbalance that impacts the photosynthetic pigments, leading to a reduction in photosynthetic activities (Ali et al., 2013). At the physiological level, leaf water potential, therefore, is influenced by salinity stress. It creates a water deficit in plants, forcing stomatal closure and a decline in gas exchange processes with an increased probability of oxidative damage (Abideen et al., 2020). Reduced photosynthesis can be inhibited by decreased gas exchange due to stomatal closure (Abideen et al., 2020). Plant types vary in salinity tolerance, with halophytes being more adaptable under saline conditions than glycophytes (Flowers and Colmer, 2015). In glycophytic crops, salinity tolerance involves maintaining ion homeostasis by managing Na^+^/K^+^ ratios and excluding Na^+^ ions (Blumwald, 2000). Additionally, these plants accumulate compatible solutes such as proline, which function as osmoprotectants to stabilize protein structures and mitigate osmotic stress under salinity stress (Zulfiqar et al., 2019). Inherent antioxidant enzymes like superoxide dismutase (SOD), ascorbate peroxidase (APX), catalase (CAT), and glutathione reductase (GR) help protect against ROS toxicity (Singh et al., 2022b). However, long-term salinity stress diminishes these adaptive mechanisms and hampers the plant’s morphological, physiological, and biochemical processes. As a result, the plant cannot survive long-term stress and ultimately dies, leading to reduced crop production. This decline negatively impacts the United Nations’ sustainable development goal of “Zero Hunger” by 2030 (Rehman et al., 2023).

Scientific researchers are exploring new methods for managing saline soils and improving crop resistance to salt stress. Nanotechnology has become a promising approach, as nanoparticles (NPs) enhance nutrient use efficiency in plants (Shoukat et al., 2024). Their unique properties- such as small size, adaptable shape, and high surface area-to-volume ratio- make NPs effective carriers for agrochemicals, providing significant agricultural advantages (El-Bendary et al., 2024). Nanofertilizers (NFs) are a source of nanonutrients available at the nano, micro, and macro scales, in powder or liquid form, with a diameter of 100 nm or less, to promote plant growth (Chhipa, 2017). By increasing nutrient availability, they enhance both plant growth and yield. In summary, NFs are: (i) they supply sufficient nutrients required to enhance plant growth through foliar and soil applications; (ii) they are low-cost and long-term suppliers of plant nutrients due to higher solubility compared with conventional fertilizers; and (iii) they play a key role in reducing the abiotic stress load on plants (Adelere and Lateef, 2016). The dual application approach of NFs (i) nutrient use efficiency (NUE), (ii) abiotic stress management, promoted the development of new formulated NFs fertilizers. Nutrients in plants are vital for osmotic regulation, cellular permeability, and serve as structural components and essential metabolites, crucial for growth and development (Kulcheski et al., 2015). Among the macronutrients, nitrogen (N), phosphorus (P), and potassium (K) and micronutrients like iron (Fe), zinc (Zn), magnesium (Mg), bromine (Br), copper (Cu) and molybdenum (Mo) are often added as fertilizers in agriculture due to their importance as limiting factors (Kulcheski et al., 2015). Previously, different studies for crops show that application of NFs can reduce the plant stress and help with NUE. For example, Nano-NPK with a combination of micronutrient treatment enhances the overall plant health of wheat through various interconnected mechanisms (Ding et al., 2025). It promotes growth, with increases in plant height (55.81%), leaf length (120.28%), and leaf area (527.25%). Biomass accumulation is also boosted, showing improvements in root dry weight (176.96%) and shoot dry weight (71.52%). Physiologically, it elevates photosynthetic capacity with higher levels of chlorophyll a (93.5%), chlorophyll b (145.6%), and carotenoids (351.7%), also improving proline and antioxidant enzyme activities that reduced the oxidative marker MDA content. Water relations improve as relative water content increases by 61.35%, supporting better cellular functions (Ding et al., 2025). NFs developed through innovative nano-chelating technology significantly enhance crop production quality and quantity (Fakharzadeh et al., 2020). A experiment assessed the effects of four micronutrient nanoparticles (NPs): zinc oxide (ZnO), silicon dioxide (SiO_2_), titanium dioxide (TiO_2_), and ferric oxide (Fe_2_O_3_) at a concentration of 50 mg/L on linseed morphology and physiology under NaCl stress (Singh et al., 2021). Treated plants demonstrated improved growth and nutrient assimilation, while salt stress elevated proline, hydrogen peroxide, and superoxide anion levels. The application of NPs enhanced the antioxidant enzymatic system and overall physiological responses, indicating increased growth, physiology, and salt tolerance in linseed plants (Singh et al., 2021). Another study investigates the impact of foliar application of Nano NPK fertilizer (19:19:19) on the growth of *Coleus aromaticus* Benth. (commonly known as Ajwain Patta) (Akhter et al., 2025). Three concentrations (2, 4, and 6 g L^−1^) were tested, revealing significant enhancements in growth traits and physiological parameters. The highest concentration (6 g L^−1^) led to a 31.4% increase in nitrate reductase activity, as well as significant increases in total phenolic (49.1%) and flavonoid content (51.7%) (Akhter et al., 2025). Additionally, essential oil content and yield saw increases of 140% and 234%, respectively, indicating that Nano NPK effectively boosts secondary metabolism in medicinal plants. They demonstrate superior solubility compared to traditional fertilizers and have a strong absorption capacity across a wide pH range (3 < pH < 11) (Harsinia et al., 2014). These fertilizers provide a gradual release of essential nutrients, reducing waste and soil pollution associated with conventional fertilizers.

To the best of our knowledge, the current study is among the few that focus on evaluating the effects of fully nanochelated fertilizer formulations on crop performance under salinity stress. Hence, this work examines the effects of three nanochelated fertilizers, including potassium-rich NPK (12-12-36), balanced NPK (20–20–20), and a complete micronutrient chelate complex (Fe-7%, Zn-1.5%, Mg-1.5%, Br-0.5%, Cu-0.5% and Mo-0.5%), on the growth and yield performance of wheat and oats in a pot trial exposed to different levels of salinity. The study aims to evaluate their impact on plant growth, photosynthetic efficiency, proline accumulation and antioxidant enzyme activity, and grain and yield quality, to provide effective sources for increased resistance to stress factors and optimization of NUE.

## 2 Materials and methods

### 2.1 Description of experimental conditions and treatment design

**Figure 1:**
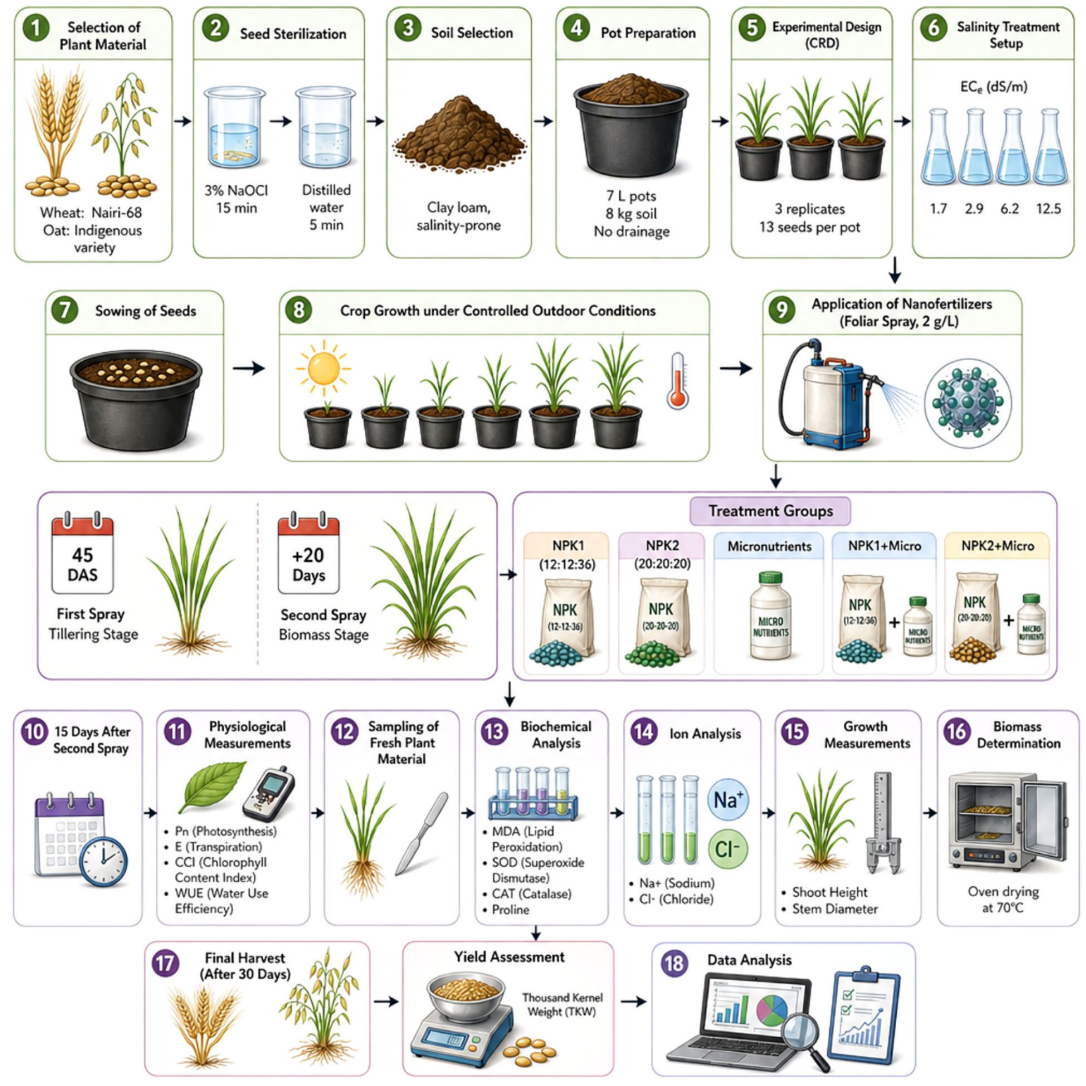
Experimental layout for the nano-chelating experiment on wheat and oat cultivation management under salinity stress.

Experiments were carried out at the experimental field of Yerevan State University, Republic of Armenia (coordinates: 40° 10′ 57.85″ N, 44° 31′ 36.97″ E). The site is located at an altitude of approximately 1040 meters above sea level and is characterized by a dry continental climate, with very dry and warm summers and mild to moderately cold winters. Armenian genotypes of wheat (*Triticum aestivum* L., Nairi-68) and oat (*Avena sativa* L., indigenous variety) were selected for the study (**Figure 1**). Plant seeds were obtained from the seed reserve fund of the National Agrarian University of Armenia. Before sowing, the seeds were disinfected for 15 minutes in a 3% sodium hypochlorite solution, then for 5 minutes in distilled water.

The experiment was conducted under outdoor conditions, and the duration of the experiment was four months. Clay loam soil was selected because it is representative of agricultural soils of the Ararat Plain, which are affected by salinity, and has moderate water retention and permeability, allowing reliable simulation of salinity development and crop responses under controlled conditions. The soil was placed in 7 L plastic containers, each containing approximately 8 kg of dry soil. An experimental design, known as a completely randomized design (CRD), was employed, where each treatment represented three replicate pots, each of which was sown with 13 seeds. The pots were sealed at the bottom without drainage holes to prevent leaching of salts. The salinity levels in the pot soils corresponded to the international classification of the electrical conductivity of the saturated soil extract (**Table 1**). The resulting EC_e_ values were 1.7, 2.9, 6.2, and 12.5 dS m^-1^, corresponding to non, slight, moderate, and high salinity levels. All pots were protected from rainfall, and the soil surface was gently loosened after each irrigation to minimize surface crust formation.

**Table 1:** Classification of soil salinity degree according to EC_e_ (dS m^-1^).

Salinity level	Range	Explanation
Non	0–2 dS m^-1^	Salinity effects are minimal
Slight	2–4 dS m^-1^	Sensitive crops show reduced yields
Moderate	4–8 dS m^-1^	Yields of many crops are limited
High	8–16 dS m^-1^	Only some crops produce acceptable yield
Extreme	>16 dS m^-1^	Only highly salt-tolerant species survive

The experiment used three different compositions of nanochelated fertilizers of the KHAZRA, Company, which are presented in the form of a powder completely soluble in water and obtained using advanced chelate technology, ensuring high nutrient bioavailability and efficient assimilation by plants. Nanochelated fertilizers were applied as foliar sprays in the form of solutions at a concentration of 2 g/L. The first application was performed 45 days after sowing, at the tillering stage, and the second application was carried out 20 days later, during the period of intensive biomass formation.

The first version (NPK_1_) is a complex nanochelated fertilizers rich in potassium, Nano Chelated NPK (12:12:36), which contains nitrogen (N-12%), phosphorus (P_2_O_5_-12%) and potassium (K_2_O-36%). The second version (NPK_2_) is represented by a balanced composition Nano Chelated NPK (20-20-20) fertilizer, which contains nitrogen, phosphorus and potassium in an equal ratio (20:20:20). The third option (Micronutrients) is a complex of micronutrients - Nano Chelated Complete Micro Fertilizer, which contains iron (Fe-7%), zinc (Zn-1.5%), magnesium (Mg-1.5%), bromine (Br-0.5%), copper (Cu-0.5%) and molybdenum (Mo-0.5%). The experimental scheme provided for both separate and combined applications, in particular, NPK_1_ and micronutrients, and NPK_2_ and micronutrients options, with the aim of evaluating the combined effect of macro- and micronutrients on plant growth, morpho-physiological and biochemical activity and salt tolerance.

After 15 days of the second treatment with nanochelated fertilizers, the physiological indices of the crops were measured: photosynthetic rate (Pn), transpiration rate (E), and chlorophyll content index (CCI). And 3 plants were collected from each pot to determine the activity of enzymes, since enzymes are sensitive proteins that retain their structure and activity only in living, water-rich and metabolically active cells. In the event of drying or yellowing of the leaf, these proteins are denatured, which leads to a complete loss of enzyme activity and makes accurate measurement impossible. Then, after 30 days, the crops were harvested for further studies.

### 2.2 Determination of growth and biomass attributes

#### 2.2.1 Measurement of shoot height and stem diameter

Wheat and oat genotypes were assessed using two primary morphological parameters: shoot height and stem diameter. The results are presented as mean values. Shoot height was determined from the soil surface to the apical point of the plant. Stem diameter was measured immediately after harvest using a Vernier caliper, at a point located approximately 2 cm above the root-shoot junction along the basal stem (Ghazaryan et al., 2023).

#### 2.2.2 Measurement of biomass

Plant biomass (dry) was determined at harvest maturity, when the plants were finally harvested (Ding et al., 2025). The aboveground part of the plant was collected, separated from the seeds, first washed with tap water, then rinsed with distilled water, after which the samples were dried for about 72 hours in a constant temperature drying oven (Biobase BJPX-HDO138, PRC) at 70 °C until a constant dry mass was obtained.

### 2.3 Determination of ions concentrations

Dried shoots of the plants were ground into a fine powder and digested in a 0.5% HNO_3_ solution at 95-100 °C for 30 minutes to extract soluble ions. The digestion was carried out using 1.00 g of dry plant material in 100 mL of the acid solution. Following digestion, the extracts were filtered through filter paper and analyzed immediately. The concentration of sodium (Na^+^) was determined using a flame photometer (FP-I6431, Bioevopeak, PRC), and the concentration of Cl^-^ was measured using a laboratory ionometer (I-160 M, Anatech, Belarus). Each determination was performed in five replicates per sample to ensure analytical accuracy (Margaryan et al., 2025).

### 2.4 Determination of photosynthesis indices

#### 2.4.1 Estimation of chlorophyll content index

Chlorophyll content was measured as an index of the physiological response of plants growing under saline soil conditions. The measurements were conducted on fully expanded leaves from the upper part of the plants using a CCM-200 Plus Chlorophyll Content Meter (Opti-Sciences, USA). For each plant, ten readings were taken at different points on the leaf surface, and the mean chlorophyll content index (CCI) was automatically calculated by the device.

CCI is calculated using the following Equation 1:

(1) $$CCI=\frac{\%T_{931}}{\%T_{653}}$$

where %T_931_ and %T_653_ represent the leaf spectral transmittance values measured at wavelengths of 931 nm and 653 nm, respectively. The wavelength of 653 nm corresponds to the major absorption peak of chlorophyll pigments in the red region of the visible spectrum, while 931 nm lies in the near-infrared region with minimal chlorophyll absorption. This ratio provides a rapid and non-destructive estimate of relative chlorophyll content in leaf tissues, where higher CCI values indicate greater chlorophyll concentration (Silla et al., 2010).

#### 2.4.2 Gas exchange parameters

The net photosynthetic rate (Pn) and transpiration rate (E) were measured using a portable photosynthesis system CI-340 (CID Bio-Science, USA) under the following environmental conditions: air pressure between 89.55 and 89.90 kPa, air temperature within 25-30 °C, and ambient CO_2_ concentration ranging from 390 to 405 μmol mol^-^¹. Measurements were performed on one young, fully expanded leaf from each plant, between 08:00 and 11:00.

Water use efficiency (WUE) was expressed as the net carbon uptake per amount of water lost through transpiration and calculated according to the following relationship as Equation 2 (Rabhi et al., 2012):

(2) $$WUE=\frac{Pn}{E}$$

where Pn is the net photosynthetic rate (μmol CO_2_ m^-^² s^-^¹) and E is the transpiration rate (mmol H_2_O m^-^² s^-^¹).

### 2.5 Lipid peroxidation and enzymatic antioxidants indices

#### 2.5.1 Determination of MDA content

Lipid peroxidation was assessed following a modified TBA procedure described in recent methodologies (Ali et al., 2024; Meftahizadeh et al., 2025). Fresh biomass was homogenized in Tris-HCl buffer (0.025 M, pH 7.4), treated with 17% TCA, and clarified by centrifugation. The supernatant was heated with 0.8% TBA (95-100 °C, 10 min). To ensure precision in plant extracts, the 600 nm absorbance was subtracted from the 532 nm reading to eliminate background interference (Hodges et al., 1999). The final MDA concentration was calculated using the 1.56 × 10^5^ M^-^¹ cm^-^¹ extinction coefficient (Heath and Packer, 1968).

#### 2.5.2 Determination of superoxide dismutase activity

For SOD determination, fresh biomass was homogenized separately in an extraction matrix of 0.25 M sucrose and 1 mM EDTA and clarified by centrifugation. The suppression of epinephrine autoxidation was continuously monitored at 347 nm. The test mixture (pH 10.65 bicarbonate buffer, sample, and epinephrine hydrochloride) was analyzed at 30-second intervals for 3 min. The 50% suppression threshold was established as one standard unit of SOD activity (Volkov et al., 2023).

#### 2.5.3 Determination of catalase activity

Samples were mechanically disrupted in an ice-cold extraction medium consisting of 50 mM phosphate buffer (pH 7.4) and 1 mM EDTA. Following centrifugation, the functional assay involved combining the active supernatant with H_2_O_2_ for 3 min at 37 °C. Ammonium molybdate was applied to quench the reaction, and the unreacted complex was quantified at 374 nm (Hadwan and Abed, 2016).

#### 2.5.4 Determination of free proline

Both endogenous free proline and proline biosynthetic enzyme activity were evaluated using a modified ninhydrin colorimetric assay (Bates et al., 1973; Rabbi et al., 2025). Tissues were homogenized in Na-K phosphate buffer (pH 7.4) and centrifuged to yield a cell-free extract. To determine the free proline pool, a fraction of the baseline extract was directly combined with a ninhydrin reagent and heated at 95-100 °C for 15 min. After rapid cooling, optical density was measured at 520 nm, and proline content was derived using an L-proline standard curve.

### 2.6 Yield-related parameters

At the end of the experiment, a certain number of seeds were obtained for each treatment. In order to ensure the comparability of the obtained data, a recalculation was performed based on the Thousand Kernel Weight (TKW) (Wu et al., 2018). TKW is an integral indicator of seed number and mass. It is used to standardize the amount of seed material between different treatments, as well as to ensure a correct comparison of biological and productive indicators (Wu et al., 2018).

### 2.7 Statistical analysis

Statistical analyses were performed using GraphPad Prism software, version 8.0 (GraphPad Software, San Diego, CA, USA). Significant differences among treatments were determined by two-way ANOVA followed by Fisher’s LSD test at P < 0.05.

## 3 Results

### 3.1 Shoot growth and biomass

#### 3.1.1 Shoot height and stem diameter

**Figure 2:**
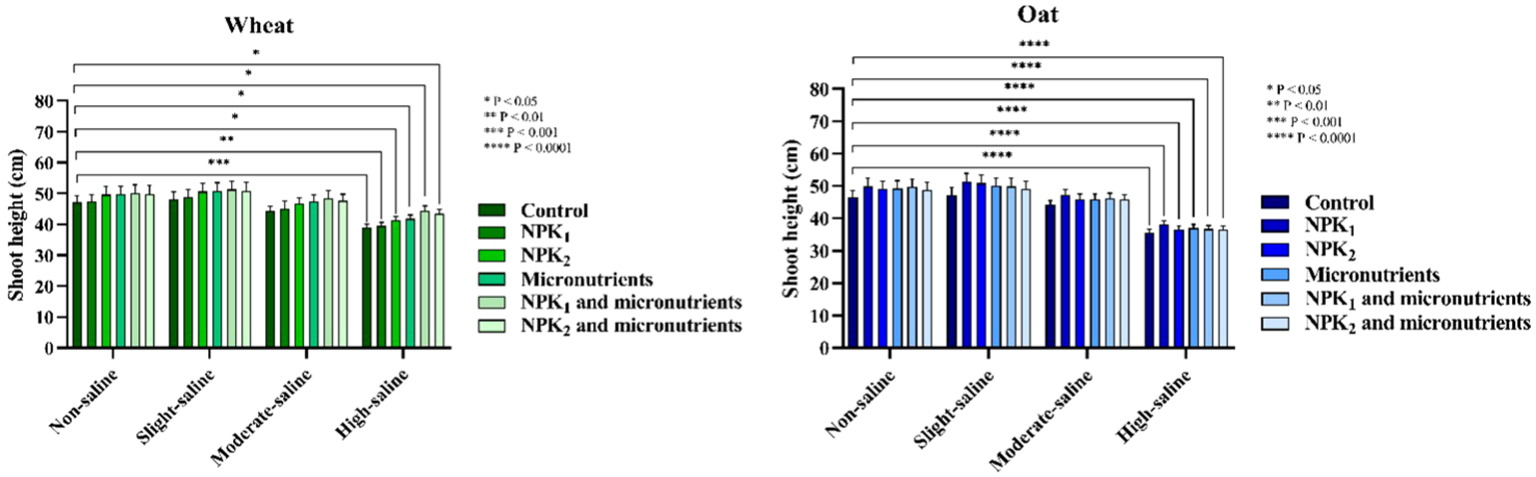
Effect of nanochelated fertilizers on shoot height under salinity stress. The data in the figure represent means ± standard errors, and the values are means of three replicates with ten plants per replicate. Significant differences (p ≤ 0.05) are marked with asterisks. *Significant difference at p < 0.05; **significant difference at p < 0.01; ***significant difference at p < 0.001; and ****significant difference at p < 0.0001.

The shoot heights of the studied crops underwent various changes, both with increasing salinity and as a result of different nanochelated fertilizer treatments (**Figure 2**). In particular, in the case of wheat, shoot height in the Slight-saline Control increased slightly (2.0%) compared to the Non-saline Control, and then decreased to 17.4% in the High-saline Control. At all salinity levels, nanochelated fertilizer treatments had a positive effect on shoot height. In particular, the more effective result was observed in the combination of NPK_1_ and micronutrients treatment, where shoot height under High-saline NPK_1_ and micronutrients treatment decreased by 5.8% compared to the Non-saline Control.

In the case of oats, also with increasing salinity, compared to the Non-saline Control, an increase in shoot height was observed in the Slight-saline Control, amounting to 1.5%, then it decreased and amounted to 23.5% in the High-saline Control. Similarly, across all salinity levels, nanochelated fertilizers had a positive effect on plant height growth. In particular, the surpassing result was recorded in the case of NPK_1_ treatment, compared to the Non-saline Control, the percentage of decrease in the High-saline NPK_1_ treatment was 17.8%.

**Figure 3:**
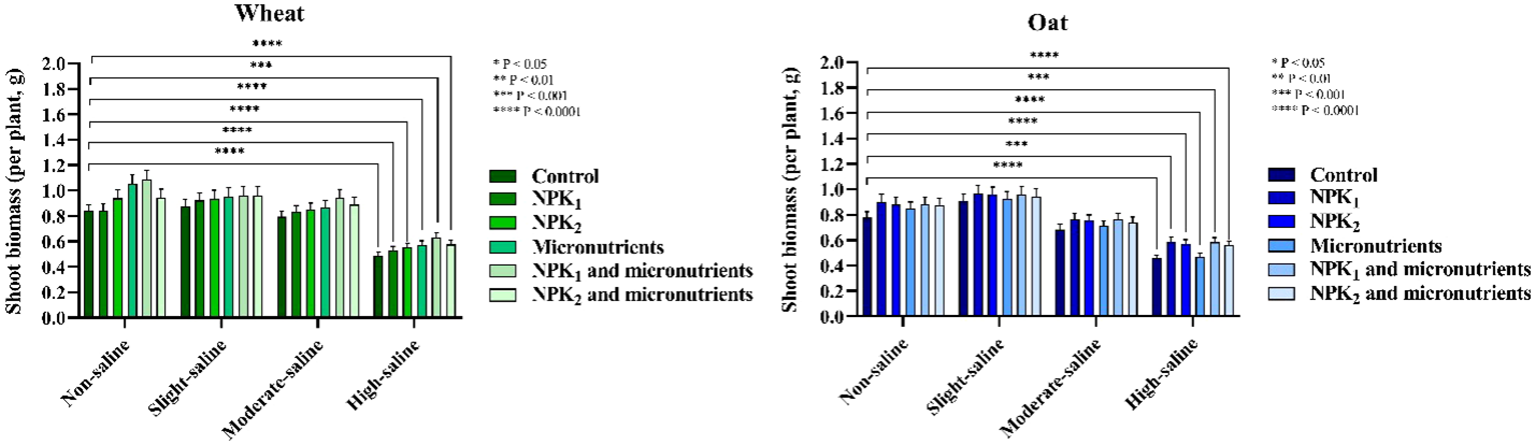
Effect of nanochelated fertilizers on stem diameter under salinity stress. The data in the figure represent means ± standard errors, and values are means of three replicates with ten plants per replicate. Significant differences (p ≤ 0.05) are marked with asterisks. *Significant difference at p < 0.05; **significant difference at p < 0.01; ***significant difference at p < 0.001; and ****significant difference at p < 0.0001.

The effect of nanochelated fertilizers on stem diameter under salt stress conditions is presented in **Figure 3**. In the case of wheat, as salinity increased, stem diameter decreased by 7.0% in the High-saline Control compared to the Non-saline Control. As in the case of plant height, nanochelated fertilizers had a positive effect on stem diameter growth at all salinity levels. In particular, the more significant result was recorded in the case of the combination of NPK_1_ and micronutrients treatment, compared to the Non-saline Control, the percentage of decrease in the High-NPK_1_ and micronutrients treatment was 4.2%.

In the case of oats, as salinity increased, the stem diameter decreased by 17.7% in the High-saline Control compared to the Non-saline Control. The significant effect of the nanochelated fertilizer was recorded in the case of NPK_1_ treatment, compared to the Non-saline Control, the percentage of decrease in the High-saline NPK_1_ treatment was 11.9%.

#### 3.1.2 Shoot biomass

**Figure 4:**
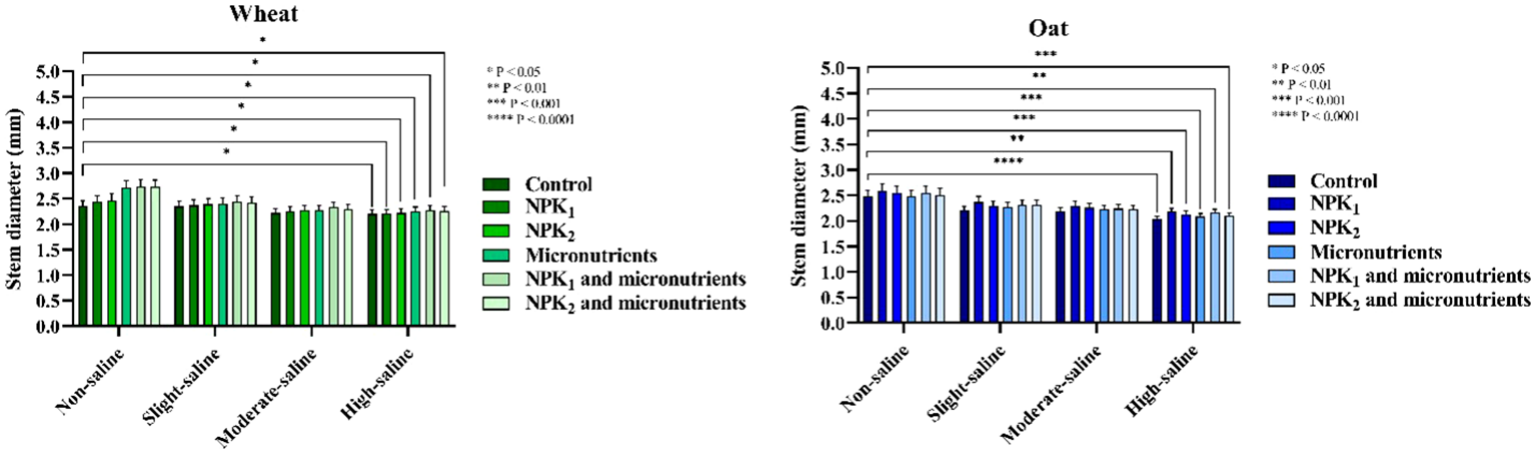
Effect of nanochelated fertilizers on shoot biomass (dry) under salinity stress. The data in the figure represent means ± standard errors, and the values are means of three replicates with ten plants per replicate. Significant differences (p ≤ 0.05) are marked with asterisks. *Significant difference at p < 0.05; **significant difference at p < 0.01; ***significant difference at p < 0.001; and ****significant difference at p < 0.0001.

Assessment of shoot biomass indicators allows us to identify the effects of salt stress and nanochelated fertilizer application on plant growth and activity intensity **Figure 4** shows the changes in the average biomass of crops as a result of nanochelated fertilizer application under salt stress conditions. In the case of wheat, as salinity increased, biomass increased in the Slight-saline Control compared to the Non-saline Control by 4.5%, then began to decrease and was 41.8% in the High-saline Control. The effect of nanochelated fertilizers on biomass was also positive, here too the surpassing result was recorded in the case of the combination of NPK_1_ and micronutrients treatment, compared to the Non-saline Control, the percentage of decrease in the High-saline NPK_1_ and micronutrients treatment was 25.0%.

In the case of oats, also with increasing salinity, compared to the Non-saline Control, an increase in biomass was observed in the Slight-saline Control, amounting to 16.6%, then it decreased and amounted to 41.0% in the High-saline Control. The effect of nanochelated fertilizers on shoot biomass was again positive, in particular, the more effective result was recorded in the case of NPK_1_ treatment, compared to the Non-saline Control, the percentage of decrease in the High-saline NPK_1_ treatment was 24.5%.

### 3.2 Salt accumulation attributes

**Figure 5:**
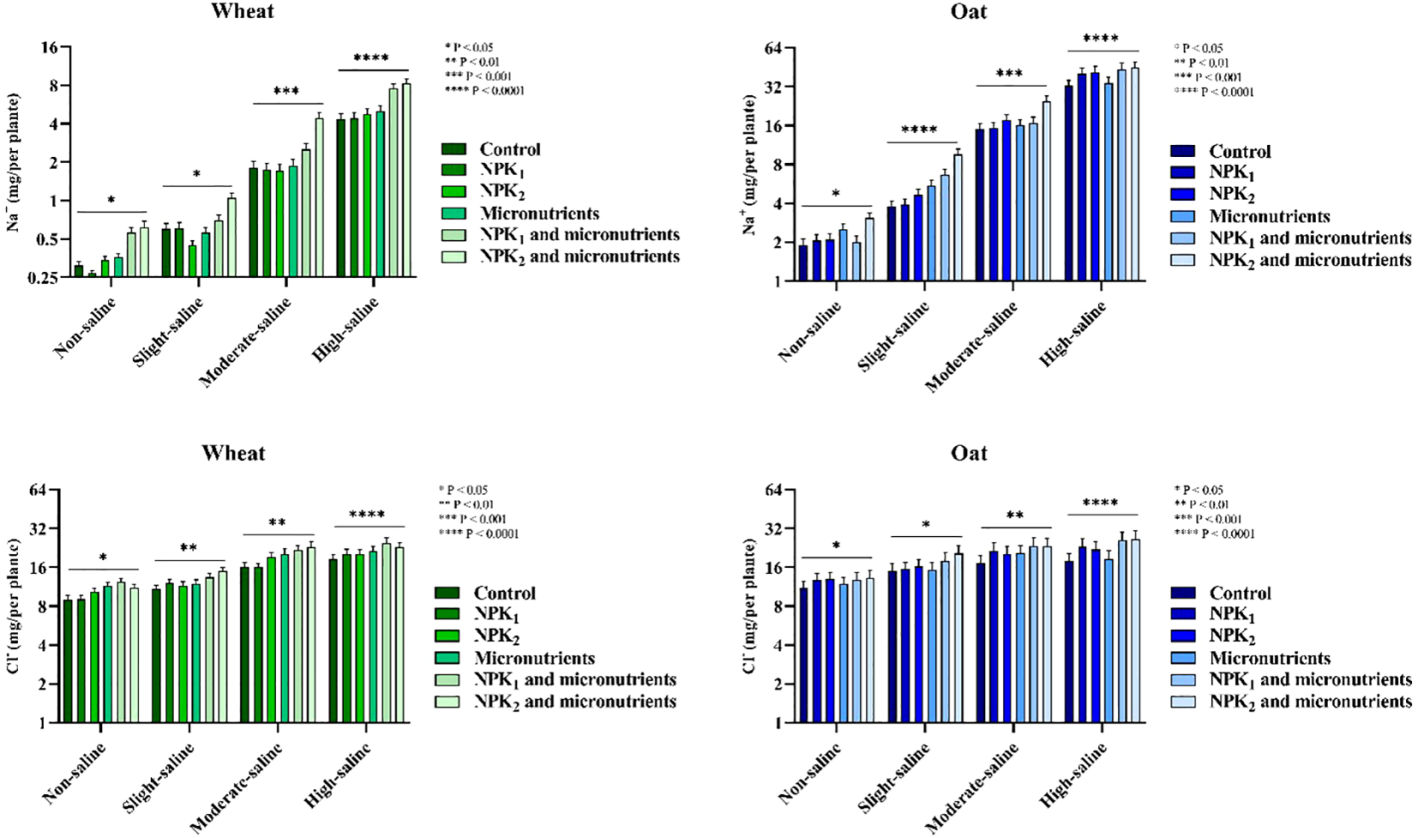
The mass of Na^+^ and Cl^-^ (mg) accumulated in the aboveground parts of the studied crops per plant. The data in the figure represent means ± standard errors, and the values are means of three replicates with ten plants per replicate. Significant differences (p ≤ 0.05) are marked with asterisks. *Significant difference at p < 0.05; **significant difference at p < 0.01; ***significant difference at p < 0.001; and ****significant difference at p < 0.0001.

The accumulation of Na^+^ and Cl^-^ ions in the aboveground tissues of crops under salt stress, in response to nanochelated fertilizer application, is presented in **Figure 5**. In both crops, with increasing soil salinity, an overall increase in the ion content is observed, which indicates enhanced salt uptake and transport under stress conditions. In the case of wheat, Na^+^ accumulation gradually increased and reached its maximum under conditions of high salinity, while the Cl^-^ content per plant remained relatively stable, showing only minor fluctuations. With increasing salinity, an increase in the Na^+^ and Cl^-^ contents is observed, but at the same time a decrease in the aboveground mass is also observed. As a result, the total Cl^-^ content in the aboveground mass of one plant almost does not change, while in the case of Na^+^, the increase in the content exceeds the decrease in biomass, due to which its amount in the aboveground part of one plant increases significantly. It should be noted that as a result of the application of nanochelated fertilizers, the concentrations of Na^+^ and Cl^-^ ions mainly increased.

### 3.3 Photosynthetic attributes

#### 3.3.1 Chlorophyll content index

**Figure 6:**
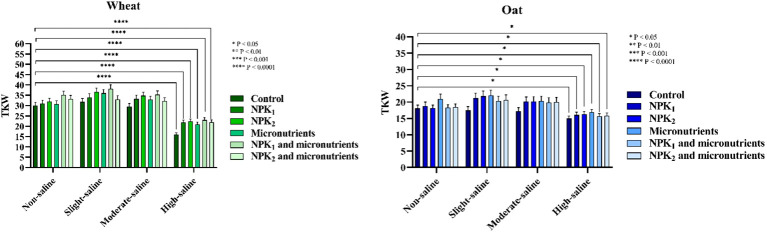
Effect of nanochelated fertilizers on CCI under salinity stress. The data in the figure represent means ± standard errors, and the values are means of three replicates with thirteen plants per replicate and ten measurements per plant. Significant differences (p ≤ 0.05) are marked with asterisks. *Significant difference at p < 0.05; **significant difference at p < 0.01; ***significant difference at p < 0.001; and ****significant difference at p < 0.0001.

Chlorophyll content index (CCI) is an important physiological indicator of plant status and photosynthetic activity, reflecting changes in the pigment system under environmental influences. Changes in CCI under the influence of nanochelated fertilizers in the studied crops under salt stress are presented in **Figure 6**. In the case of wheat, as salinity increased, the CCI value in the Slight-saline Control increased by 9.6% compared to the Non-saline Control, then began to decrease and amounted to 23.0% in the High-saline Control. The effect of nanochelated fertilizers on the CCI value also had a positive effect; the most effective result was recorded in the case of NPK_2_ treatment, compared to the Non-saline Control. The percentage of decrease in the High-saline NPK_2_ treatment was 12.5%. Under high salinity conditions, when comparing the control variant (High-saline Control) with plants treated with NPK_2_ nanochelated fertilizers (High-saline NPK_2_ treatment), it was recorded that the CCI value increased by 13.6% in the latter variant, which indicates a more positive effect of NPK_2_ application.

In the case of oats, as salinity increased, the CCI value increased again in the Slight-saline Control compared to the Non-saline Control, amounting to 8.6%, and then began to decrease and amounted to 51.9% in the High-saline Control. The effect of nanochelated fertilizers on the CCI value was positive, and as with wheat, the surpassing result was recorded in the case of NPK_2_ treatment. Compared to the Non-saline Control, the percentage of decrease in the High-saline NPK_2_ treatment was 27.0%. And when we compare the High-saline Control with High-saline NPK_2_ treatment, the CCI value of the latter increased by 51.8%, which is a significantly positive result.

#### 3.3.2 Gas exchange parameters

Changes in gas exchange parameters under the influence of nanochelated fertilizers and salt stress are presented in **Table 2**. In the studied crops, Pn values ​​decreased with increasing salinity, but at each salinity level, an increase in photosynthesis intensity was recorded as a result of treatment with nanochelated fertilizers. In the case of wheat, the Pn value gradually decreased, and compared to the Non-saline Control, the percentage of decrease in the High-saline Control was 54.6%. The more significant result from nanochelated fertilizers was obtained in the case of the combination of NPK_2_ and micronutrients treatment, compared to the Non-saline Control, the decrease in the High-saline NPK_2_ and micronutrients treatment was 17.9%.

**Table 2:** Effect of nanochelated fertilizers on gas exchange parameters under salinity stress.

Crop	Salinity degree	Treatment	Pn (μmol CO_2_ m^-^² s^-^¹)	E (mmol H_2_O m^-^² s^-^¹)	WUE
Wheat	Non-saline	Control	5.64 ± 0.58	2.08 ± 0.21	2.72 ± 0.28
		NPK_1_	5.95 ± 0.60	2.16 ± 0.22	2.75 ± 0.28
		NPK_2_	5.71 ± 0.58	2.09 ± 0.21	2.73 ± 0.27
		Micronutrients	6.28 ± 0.63	2.31 ± 0.24	2.72 ± 0.29
		NPK_1_ and micronutrients	6.29 ± 0.63	2.29 ± 0.24	2.75 ± 0.28
		NPK_2_ and micronutrients	6.30 ± 0.64	2.12 ± 0.22	2.97 ± 0.30
	Slight-saline	Control	4.93 ± 0.50	1.24 ± 0.11	3.99 ± 0.40
		NPK_1_	5.75 ± 0.59	1.31 ± 0.13	4.39 ± 0.45
		NPK_2_	5.30 ± 0.55	1.29 ± 0.12	4.10 ± 0.43
		Micronutrients	6.58 ± 0.67	1.51 ± 0.16	4.36 ± 0.44
		NPK_1_ and micronutrients	6.73 ± 0.68	1.62 ± 0.18	4.16 ± 0.42
		NPK_2_ and micronutrients	6.80 ± 0.70	1.34 ± 0.15	5.09 ± 0.52
	Moderate-saline	Control	4.25 ± 0.44	1.05 ± 0.11	4.05 ± 0.41
		NPK_1_	4.55 ± 0.46	1.11 ± 0.12	4.11 ± 0.42
		NPK_2_	4.45 ± 0.46	1.09 ± 0.10	4.07 ± 0.42
		Micronutrients	4.62 ± 0.47	1.06 ± 0.10	4.35 ± 0.45
		NPK_1_ and micronutrients	4.66 ± 0.47	1.14 ± 0.13	4.08 ± 0.41
		NPK_2_ and micronutrients	4.70 ± 0.49	1.11 ± 0.12	4.22 ± 0.43
	High-saline	Control	2.56 ± 0.27	0.53 ± 0.06	4.85 ± 0.50
		NPK_1_	4.05 ± 0.41	0.73 ± 0.08	5.52 ± 0.56
		NPK_2_	3.54 ± 0.36	0.60 ± 0.05	5.93 ± 0.60
		Micronutrients	4.35 ± 0.45	0.72 ± 0.08	6.05 ± 0.62
		NPK_1_ and micronutrients	4.54 ± 0.48	0.87 ± 0.09	5.24 ± 0.55
		NPK_2_ and micronutrients	4.63 ± 0.47	0.79 ± 0.08	5.60 ± 0.58
Oat	Non-saline	Control	4.18 ± 0.42	0.89 ± 0.10	4.72 ± 0.49
		NPK_1_	4.29 ± 0.44	0.73 ± 0.07	5.88 ± 0.60
		NPK_2_	4.65 ± 0.48	0.82 ± 0.09	5.68 ± 0.58
		Micronutrients	4.50 ± 0.47	0.75 ± 0.07	6.03 ± 0.61
		NPK_1_ and micronutrients	4.45 ± 0.45	0.71 ± 0.06	6.29 ± 0.63
		NPK_2_ and micronutrients	4.60 ± 0.47	0.73 ± 0.07	6.28 ± 0.63
	Slight-saline	Control	5.53 ± 0.58	1.07 ± 0.10	5.19 ± 0.52
		NPK_1_	5.66 ± 0.57	1.09 ± 0.11	5.20 ± 0.54
		NPK_2_	6.74 ± 0.68	1.19 ± 0.12	5.66 ± 0.59
		Micronutrients	5.74 ± 0.58	1.81 ± 0.19	3.17 ± 0.32
		NPK_1_ and micronutrients	6.80 ± 0.70	1.89 ± 0.20	3.60 ± 0.35
		NPK_2_ and micronutrients	6.92 ± 0.71	1.81 ± 0.19	3.83 ± 0.39
	Moderate-saline	Control	2.18 ± 0.22	0.44 ± 0.03	5.00 ± 0.49
		NPK_1_	3.41 ± 0.35	0.50 ± 0.04	6.83 ± 0.70
		NPK_2_	3.79 ± 0.39	0.58 ± 0.06	6.51 ± 0.68
		Micronutrients	3.75 ± 0.39	0.68 ± 0.07	5.50 ± 0.57
		NPK_1_ and micronutrients	3.35 ± 0.35	0.74 ± 0.07	4.53 ± 0.46
		NPK_2_ and micronutrients	3.74 ± 0.38	0.77 ± 0.08	4.83 ± 0.49
	High-saline	Control	2.13 ± 0.22	0.53 ± 0.04	4.03 ± 0.41
		NPK_1_	3.17 ± 0.32	0.61 ± 0.05	5.21 ± 0.53
		NPK_2_	3.76 ± 0.39	0.77 ± 0.08	4.87 ± 0.50
		Micronutrients	3.02 ± 0.31	0.69 ± 0.07	4.41 ± 0.46
		NPK_1_ and micronutrients	3.30 ± 0.35	0.75 ± 0.07	4.39 ± 0.46
		NPK_2_ and micronutrients	3.66 ± 0.38	0.79 ± 0.08	4.66 ± 0.48

The data in the table represent means ± standard errors and values are means of three replicates with thirteen plants per replicate (P< 0.05).

In the case of oats, the Pn value increased initially and then decreased sharply. Compared to the Non-saline Control, the Pn value in the Slight-saline Control increased by 32.4%, while the percentage decrease in the High-saline Control was 49.1%. The more appropriate result among the nanochelated fertilizers was obtained in NPK_2_ treatment, except for the slight salinity level, where the surpassing result was in the case of the combination of NPK_2_ and micronutrients treatment. Compared to the Non-saline Control, the decrease in the High-saline NPK_2_ treatment was 10.0%.

In wheat, the E value decreased with increasing salinity. Compared to the Non-saline Control, the percentage reduction in the *High-Saline Control* was 74.5%. The positive result from the nanochelated fertilizers was obtained with the combination of NPK_1_ and micronutrients treatment. Compared to the Non-saline Control, the reduction in the High-saline NPK_1_ and micronutrients treatment was 58.3%.

In the case of oats, the E value initially increased and then decreased with increasing salinity. Compared to the Non-saline Control, the E value in the Slight-saline Control increased by 20.3%, while the percentage decrease in the High-saline Control was 40.6%. The positive result from the nanochelated fertilizers was obtained with the combination of NPK_2_ and micronutrients treatment, except for the slight salinity level, where the prominent value was with the combination of NPK_1_ and micronutrients treatment. Compared to the Non-saline Control, the decrease in the High-saline NPK_2_ and micronutrients treatment was 11.4%.

In the case of wheat, the WUE value increased with increasing salinity. Compared to the Non-saline Control, the percentage increase in the High-saline Control was 78.3%. The more appropriate result from the nanochelated fertilizers was obtained in the case of micronutrient treatment, except in the Slight-saline group, where the highest value was in the case combination of NPK_2_ and micronutrient treatment. In the case of oats, the WUE value increased by 14.4% in the High-saline Control compared to the Non-saline Control, and no significant changes were observed in the nanochelated fertilizers treatment options.

### 3.4 Lipid peroxidation and enzymatic antioxidants activities attributes

#### 3.4.1 MDA content

MDA content is an important biochemical indicator of oxidative stress and the extent of cell membrane damage (**Table 3**). In the case of wheat, as salinity increased, MDA values ​​increased. Compared to the Non-saline Control, it increased 4.11 times in the High-saline Control. Under the influence of nanochelated fertilizers, MDA values ​​decreased compared to the control groups at all salinity levels, with the lowest value recorded in the case of the combination of NPK_2_ and micronutrients treatment. Compared to the High-saline Control, the value decreased 1.29 times in the High-saline NPK_2_ and micronutrients treatment.

**Table 3:** Effect of nanochelated fertilizers on enzyme activities under salinity stress.

Crop	Salinity degree	Treatment	Amount of MDA (nmol//mg protein)	Amount of free proline (µmoles/mg protein)	Catalase activity (µmol H2O2/min/mg protein)	SOD activity (U/mg protein)
Wheat	Non-saline	Control	1.80 ± 0.16	7.50 ± 0.76	3.50 ± 0.36	1.50 ± 0.16
		NPK_1_	1.60 ± 0.15	7.90 ± 0.78	3.70 ± 0.39	1.60 ± 0.17
		NPK_2_	1.50 ± 0.13	8.20 ± 0.81	3.80 ± 0.40	1.65 ± 0.18
		Micronutrients	1.40 ± 0.14	8.40 ± 0.85	4.10 ± 0.42	1.80 ± 0.19
		NPK_1_ and micronutrients	1.30 ± 0.12	8.70 ± 0.89	3.80 ± 0.39	1.70 ± 0.18
		NPK_2_ and micronutrients	1.20 ± 0.10	9.10 ± 0.92	4.40 ± 0.45	1.85 ± 0.19
	Slight-saline	Control	3.10 ± 0.32	9.50 ± 0.96	4.90 ± 0.51	1.85 ± 0.19
		NPK_1_	2.90 ± 0.28	9.90 ± 1.01	5.10 ± 0.52	2.05 ± 0.21
		NPK_2_	2.50 ± 0.23	10.30 ± 1.05	5.40 ± 0.55	2.10 ± 0.22
		Micronutrients	2.20 ± 0.19	10.60 ± 1.09	5.80 ± 0.57	2.20 ± 0.21
		NPK_1_ and micronutrients	1.90 ± 0.18	11.20 ± 1.13	6.10 ± 0.62	2.30 ± 0.24
		NPK_2_ and micronutrients	1.70 ± 0.16	11.60 ± 1.17	6.40 ± 0.65	2.35 ± 0.25
	Moderate-saline	Control	5.70 ± 0.60	12.50 ± 1.23	6.80 ± 0.67	2.45 ± 0.26
		NPK_1_	4.50 ± 0.46	12.85 ± 1.30	7.20 ± 0.71	2.55 ± 0.26
		NPK_2_	3.90 ± 0.40	13.20 ± 1.31	7.50 ± 0.76	2.60 ± 0.27
		Micronutrients	3.50 ± 0.37	13.90 ± 1.41	7.80 ± 0.77	2.70 ± 0.29
		NPK_1_ and micronutrients	3.00 ± 0.29	14.40 ± 1.45	8.20 ± 0.83	2.80 ± 0.28
		NPK_2_ and micronutrients	2.60 ± 0.25	14.90 ± 1.50	8.40 ± 0.85	2.85 ± 0.28
	High-saline	Control	7.40 ± 0.75	13.50 ± 1.34	5.50 ± 0.56	1.90 ± 0.20
		NPK_1_	6.90 ± 0.70	13.90 ± 1.40	5.90 ± 0.60	2.00 ± 0.21
		NPK_2_	6.50 ± 0.64	14.25 ± 1.43	6.15 ± 0.62	2.05 ± 0.21
		Micronutrients	6.20 ± 0.61	14.30 ± 1.44	6.50 ± 0.66	2.20 ± 0.23
		NPK_1_ and micronutrients	6.05 ± 0.60	14.70 ± 1.49	6.85 ± 0.69	2.35 ± 0.24
		NPK_2_ and micronutrients	5.75 ± 0.59	15.20 ± 1.55	7.00 ± 0.72	2.40 ± 0.25
Oat	Non-saline	Control	1.50 ± 0.14	4.60 ± 0.47	3.00 ± 0.29	1.20 ± 0.11
		NPK_1_	1.40 ± 0.13	4.80 ± 0.49	3.00 ± 0.28	1.30 ± 0.12
		NPK_2_	1.20 ± 0.11	5.10 ± 0.53	3.30 ± 0.34	1.40 ± 0.15
		Micronutrients	1.30 ± 0.12	4.90 ± 0.51	3.20 ± 0.33	1.35 ± 0.13
		NPK_1_ and micronutrients	1.10 ± 0.10	5.30 ± 0.52	3.50 ± 0.36	1.45 ± 0.15
		NPK_2_ and micronutrients	0.90 ± 0.08	5.60 ± 0.57	3.80 ± 0.39	1.50 ± 0.16
	Slight-saline	Control	2.40 ± 0.25	8.50 ± 0.84	4.20 ± 0.45	1.60 ± 0.17
		NPK_1_	2.20 ± 0.21	9.00 ± 0.89	4.50 ± 0.47	1.70 ± 0.18
		NPK_2_	1.90 ± 0.20	9.50 ± 0.96	4.90 ± 0.50	1.80 ± 0.18
		Micronutrients	2.00 ± 0.20	9.20 ± 0.93	4.60 ± 0.47	1.75 ± 0.18
		NPK_1_ and micronutrients	1.70 ± 0.18	10.00 ± 1.04	5.20 ± 0.53	1.90 ± 0.20
		NPK_2_ and micronutrients	1.40 ± 0.13	10.50 ± 1.10	5.50 ± 0.57	2.00 ± 0.21
	Moderate-saline	Control	3.40 ± 0.35	12.50 ± 1.24	5.80 ± 0.59	2.00 ± 0.21
		NPK_1_	3.10 ± 0.33	13.20 ± 1.33	6.20 ± 0.60	2.10 ± 0.22
		NPK_2_	2.70 ± 0.29	14.00 ± 1.41	6.60 ± 0.63	2.30 ± 0.24
		Micronutrients	2.90 ± 0.31	13.50 ± 1.36	6.40 ± 0.66	2.20 ± 0.23
		NPK_1_ and micronutrients	2.40 ± 0.26	14.50 ± 1.47	7.00 ± 0.69	2.40 ± 0.24
		NPK_2_ and micronutrients	2.00 ± 0.21	15.20 ± 1.54	7.40 ± 0.75	2.60 ± 0.25
	High-saline	Control	4.50 ± 0.46	15.50 ± 1.56	7.20 ± 0.74	2.40 ± 0.23
		NPK_1_	4.10 ± 0.42	16.20 ± 1.60	7.50 ± 0.76	2.50 ± 0.26
		NPK_2_	3.70 ± 0.36	17.35 ± 1.75	7.90 ± 0.80	2.60 ± 0.27
		Micronutrients	3.80 ± 0.38	16.50 ± 1.66	7.70 ± 0.78	2.55 ± 0.27
		NPK_1_ and micronutrients	3.20 ± 0.33	17.40 ± 1.73	8.20 ± 0.84	2.70 ± 0.28
		NPK_2_ and micronutrients	2.80 ± 0.27	18.00 ± 1.81	8.60 ± 0.87	2.90 ± 0.30

The data in the table represent means ± standard errors and values are means of three replicates with three plants per replicate (P< 0.05).

In the case of oats, the value of MDA increased along with the increase in salinity. Compared to the Non-saline Control, it increased 3.00 times in the High-saline Control. Under the influence of nanochelated fertilizers, MDA values ​​decreased compared to the control groups at all levels of salinity; the lowest value was also recorded in the case of the combination of NPK_2_ and micronutrients treatment. Compared to the High-saline Control, the value in the High-saline NPK_2_ and micronutrients treatment decreased by 1.61 times.

#### 3.4.2 SOD activity

SOD activity is a key component of plant stress response, ensuring rapid detoxification of superoxide radicals and mitigation of oxidative stress (**Table 3**). In the case of wheat, along with the increase in salinity, SOD activity increased, with the highest values ​​recorded in plants grown under moderate salinity conditions, both control (2.45 U/mg protein) and nanochelated fertilizers treated groups (2.55-2.85 U/mg protein). Compared to the Non-saline Control, the value in the High-saline control increased by 1.27 times. Under the influence of nanochelated fertilizers, SOD activity increased compared to the control groups at all degrees of salinity, here also the highest value was recorded in the case of the combination of NPK_2_ and micronutrients treatment. Compared to the High-saline Control, the value in the High-saline NPK_2_ and micronutrients treatment increased by 1.26 times.

In the case of oats, as with CAT activity, SOD activity also increased with increasing salinity. Compared to the Non-saline Control, the value in the High-saline Control increased 2.00 times. Under the influence of nanochelated fertilizers, SOD activity increased compared to the control groups at all degrees of salinity, here also the highest value was recorded in the case of the combination of NPK_2_ and micronutrients treatment and in high saline condition that increase was 1.21 times.

#### 3.4.3 CAT activity

CAT activity is a key component of antioxidant defense, reflecting the plant’s ability to decompose hydrogen peroxide and limit oxidative damage (**Table 3**). In the case of wheat, among the studied crops, CAT activity increased with increasing salinity. It should be noted that the maximum values ​​of CAT activity were recorded at moderate salinity, and were relatively lower at high salinity, in particularly, compared to the Non-saline Control, increased 1.94 times in the Moderate-saline Control, and 1.57 times in the High-saline Control. Under the influence of nanochelated fertilizers, CAT activity increased compared to the control groups at all salinity levels, the highest value was recorded in the case of the combination of NPK_2_ and micronutrients treatment. Compared to the High-saline Control, the value increased 1.27 times in the High-saline NPK_2_ and micronutrients treatment. In contrast to wheat, in the case of oats, CAT activity increased with increasing salinity, even under high salinity conditions. Compared to the Non-saline Control, the value in the High-saline Control increased by 2.40 times. Under the influence of nanochelated fertilizers, CAT activity increased compared to the control groups at all levels of salinity, here also the highest value was recorded in the case of the combination of NPK_2_ and micronutrients treatment and compared to the High-saline Control its value is increased 1.19 times.

#### 3.4.4 Free proline

Free proline is an osmoprotective metabolite accumulated in plants, whose level reflects the degree of plant adaptation to stress conditions (**Table 3**). In the case of wheat, as salinity increased, the value of free proline increased. Compared to the Non-saline Control, it increased 1.80 times in the High-saline Control. Under the influence of nanochelated fertilizers, the values ​​of free proline increased compared to the control groups at all salinity levels, with the highest value recorded in the case of the combination of NPK_2_ and micronutrients treatment. Compared to the High-saline Control, the value increased 1.13 times in the High-saline NPK_2_ and micronutrients treatment.

In the case of oats, as salinity increased, the value of free proline also increased. Compared to the Non-saline Control, it increased 3.37 times in the High-saline Control. Under the influence of nanochelated fertilizers, the values ​​of free proline increased again compared to the control groups at all salinity levels, with the highest value recorded in the case of the combination of NPK_2_ and micronutrients treatment. Compared to the High-saline Control, the value increased 1.16 times in the High-saline NPK_2_ and micronutrients treatment.

In general, salinity stress induced oxidative damage in both wheat and oats, as evidenced by increased MDA and proline levels, coupled with the activation of antioxidant enzymes (CAT and SOD) as part of the plant defense response. Application of nanochelated fertilizers, particularly NPK_2_ combined with micronutrients, alleviated oxidative stress by reducing MDA accumulation while enhancing antioxidant enzyme activity and maintaining adaptive osmoprotective responses.

### 3.5 Yield-related attribute

**Figure 7:**
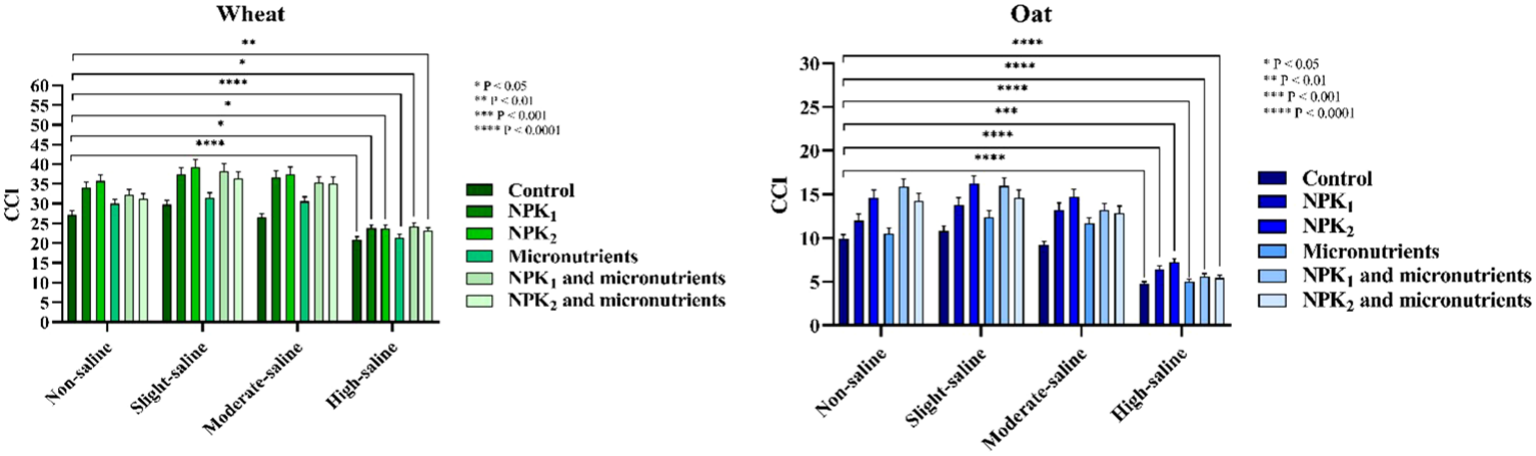
Effect of nanochelated fertilizers on seed number (TKW) under salinity stress. The data in the figure represent means ± standard errors and values are means of three replicates with ten plants per replicate. Significant differences (p ≤ 0.05) are marked with asterisks. *Significant difference at p < 0.05; **significant difference at p < 0.01; ***significant difference at p < 0.001; and ****significant difference at p < 0.0001.

The estimation of seed number based on TKW enables an integrated analysis of yield formation processes and plant responses to environmental factors (**Figure 7**). In the case of wheat, with increasing salinity, an increase in TKW was observed in the Slight-saline Control, compared to the Non-saline Control, it was 5.9%, then it started to decrease in the High-saline Control, amounting to 46.7%. Nanochelated fertilizers had a positive effect on the increase in seed number, in particular, the surpassing result was recorded in the combination of NPK_1_ and micronutrients treatment, compared to the Non-saline Control, the percentage of decrease in the High-saline NPK_1_ and micronutrients treatment was 23.3%.

In the case of oats, the TKW value decreased with increasing salinity, compared to the Non-saline Control, with a decrease of 17.1% in the High-saline Control. In both wheat and oats, the significant effect of nanochelated fertilizers was achieved with micronutrients treatment, compared to the Non-saline Control, with a decrease of 6.7% in the High-saline Micronutrients treatment.

## 4 Discussion

Wheat and oat production relies on agronomical management from sowing to harvesting. However, under salinity stress conditions, agronomical management alone is insufficient, requiring advanced technological support throughout the entire cultivation process. One such innovative solution could be the use of advanced chelate compound technology in producing high-functioning and effective fertilizers. Many studies have been designed with nano-chelating fertilizer produced by nano-chelating technology on saffron (Abdullaev et al., 2003), spinach (Moghadam et al., 2012), faba bean (E. Nadi, 2013), rice (Fakharzadeh et al., 2020), cucumber and tomato (Nazarideljou et al., 2024), and finger millet (Huq et al., 2025), indicating the beneficial and indicative effect of these fertilizers. In this study, we studied the role of nano-chelating fertilizers on improvements of morphological, physiological and biochemical parameters of wheat and oat plants under salinity stress.

### 4.1 Effect of nano-chelating fertilizers on plant growth and biomass under salinity stress

Plant growth and development is a highly complex process that is regulated by intrinsic (genetic and hormonal) factors, as well as by extrinsic (environmental or nutritional) factors through cell division, elongation and differentiation (Idrees et al., 2015). The use of nanofertilizers have been reported to improve these processes by facilitating the absorption of nutrients, meristematic activity, enzyme function and hormone biosynthesis (Mim et al., 2025). Early and late root and shoot development, therefore, are indicators of improved productivity performance, soil nutrient acquisition and efficiency of water uptake (Fang et al., 2024). A better root and shoot structure improves competitive ability in a particular set of environments (Fang et al., 2024). N, P and K are also key for promoting auxin activity while being central to different metabolic pathways, including photosynthesis as well as hormone production and a range of proteins, carbohydrates, vitamins, nucleic acids and chlorophylls (Sharma and Kumar, 2016; Metwaly, 2017). Potassium, in particular, is supportive of enzyme activity as well as stabilization of proteins and helps with the synthesis of many growth hormones, including auxin and gibberellin, by stimulating key precursors like tryptophan and geranylgeranyl diphosphate (GGDP). They induce elongation and expansion of the cells, promoting plant height, secondary stem thickness, branching, leaf development and subsequently biomass accumulation (Nofal et al., 2024). In this study, the morphological traits of wheat and oat plants, including shoot height and stem diameter, showed no significant changes at slightly saline levels; however, they declined as salinity increased from moderate to higher levels (**Figures 2**, **3**) (Ding et al., 2025; Taybi and Alyahya, 2025). Research indicates that higher NaCl concentrations negatively affect shoot height and stem diameter, which ultimately affects plant growth and biomass, particularly at high salinity levels (**Figures 2**–**4**) (Saddiq et al., 2021; Zhang et al., 2025a). The biomass of shoot was significantly reduced under higher salinity conditions, though wheat and oat plants could survive slight to moderate salinity without substantial loss in biomass (DW) (**Figure 4**) (Saddiq et al., 2021; Zhang et al., 2025a). The findings highlight the detrimental impact of salt stress on plant water status and turgor pressure, which impair biomass accumulation and lead to diminished photosynthesis rates (Ondrasek et al., 2022). Overall, the study concludes that salinity stress results in decreased biomass of wheat and oat plants, which reduces plant growth and development (**Figure 4**). Additionally, we examined the impact of foliar application of nanochelated fertilizers, including NPK_1_ (12:12:36), a balanced NPK_2_ (20:20:20), and a comprehensive micronutrient chelate complex (containing Fe-7%, Zn-1.5%, Mg-1.5%, Br-0.5%, Cu-0.5%, and Mo-0.5%) on stem height and diameter, as well as biomass. These nanochelated fertilizers promote growth in shoot height and stem diameter, leading to increased biomass in wheat and oat crops subjected to slight to moderate salinity stress. They remain effective even under higher salinity levels, with NPK_1_ and micronutrient chelate complex (in wheat), as well as NPK_1_ chelate (in oat), demonstrating particularly high efficiency in managing such conditions. These results align with previous studies on different crops and vegetables, which showed that nanochelated fertilizers (NPK and micronutrients) operate via the same mechanism to reduce stress conditions that help to improve plant growth and biomass (Janmohammadi et al., 2016; Astaneh et al., 2018; Sajyan et al., 2020; Kumar et al., 2024).

### 4.2 Balancing ionic homeostasis using nano-chelating fertilizers under salinity stress

Nutrient-deficient root zone nutrients can hinder the growth of plants in associated ion-held or nutrient-toxicity induced ionic stress. Salt stress in plants results in a limited acquisition of mineral nutrients because high concentrations of the main ions (Na^+^ and Cl^−^) inhibit nutrient uptake (Munns and Termaat, 1986). In the shoots of wheat and oat, Na^+^ and Cl^-^ concentrations increased, and previous studies also show that salinity stress further reduced Ca^2+^ and K^+^ concentrations while increasing Na^+^ and Cl^-^ concentrations (Saddiq et al., 2021; Zhang et al., 2025a). Through our experiment, we also observed negative correlations between Na^+^ and Cl^-^ content in the shoot and their growth (**Figure 5**). Furthermore, K^+^ levels in plant parts, including the shoot, are reduced with an increase of salinity both in the soil and within the plants (Abrar et al., 2022). This ionic imbalance is widely observed in plants under salinity stress or a combination of salt and water stress conditions (Abbas et al., 2018). This research shows that, across all salinity stress levels, oat shoots have the highest Na^+^ and Cl^-^ concentration following wheat shoots. The potential for Na^+^ uptake and sequestration varies widely among plant species/genotypes (Abrar et al., 2022). Uptake of Na^+^ and growth performances of wheat suggested vacuolar sequestration of Na^+^ was the most influential salt tolerance mechanism. The application of chelated NPK_1_, NPK_2_, and chelate-micronutrient-complex enhanced the tolerance due to the presence of nutritional compounds that also help in vacuolar sequestration of Na^+^ in wheat and oat plants under slight to high salinity stress levels. These amendments are beneficial for maintaining ionic balance in both crops. Similarly, previous experiments showed that using 2.5 g L^-1^ Nano-Chelate Super Plus ZFM + 5 g L^-1^ Lithovit^®^-Standard has been effective for overall nutrient uptake, which helps to increase N, P, K, and Ca concentrations significantly in both shoots and fruits. Also, this improved K^+^/Na^+^ ratio, water content, total chlorophyll and carotenoid and reduced sodium accumulation and cellular electrolyte leakage (Sajyan et al., 2020). But overall, this study concluded with our finding that nano-chelate fertilizers (NPK and micronutrients) maximized salt tolerance, which helps in avoiding the toxic effects of salinity stress.

### 4.3 Improving photosynthesis efficiency through nano-chelating fertilizers under salinity stress

Salt stress inhibited crop performance and photosystem traits such as CCI, Pn, E, and WUE (**Figure 6**; **Table 2**). Toxic Na^+^ ions in the transpiration system may cause photo-inhibition, leading to stomatal opening. Na^+^ accumulation in chloroplasts results in oxidation or denaturation of photosynthetic pigments, reducing photosynthesis efficiency by decreasing electron transport rate (ETR) and the quantum yield of photosystem II (Yang et al., 2020). Ion buildup within the photosynthesis apparatus, along with osmotic stress, indirectly reduces photosynthetic rate through physiological drought at the cellular level (Dhansu et al., 2022; Kumar et al., 2023). Additionally, under salinity stress, incomplete osmoregulation and low K^+^ uptake impair guard cell function, causing stomatal closure and directly decreasing photosynthetic performance (Kumar et al., 2016; Yun et al., 2018). Although numerous studies have reported that nano materials mitigate the effects of salinity by maintaining an appropriate water balance and potential (Lu et al., 2019). Enhancements of fluorescence, chloroplastic structures and key photosynthetic parameters in rice plants, presented a significant increase by infusion of ZnO-NPs (Faizan et al., 2021) and were also reported enhancements in wheat chlorophyll with iron-nanoparticle application at a lower dose (Singh et al., 2022a). Incorporation of chelate-micronutrient-complex could possibly be attributed to the enhanced Pn due to stabilization of the photosynthetic complex and acceleration in production of pigments, besides ameliorating ionic toxicity (**Table 2**). Maximum leaf greenness in our studies was also observed with the application of nano-chelate fertilizers (NPK and micronutrients), gradually decreasing under salt stress as identified by the CCI value due to salinity-induced leaf senescence (**Figure 6**). Under salinity stress, considerable accumulation of green pigment was observed in both wheat and oat leaves with N substitution via chelate-nitrogen (NPK_1_ and NPK_2_). The chelate-micronutrient-complex formulation supplied essential nutrients with a long duration of stay and slow release at the target site, thus contributing to the stay-green trait under salinity stress, as indicated in **Figure 6**. Most plants implement osmoregulation by biosynthesizing compatible osmolytes, which is a high-energy process (10-fold) and becomes more demanding with increasing severity of salinity stress. Further, the exogenous application of nano-N may alleviate salinity-induced detrimental consequences to some extent, with continuous nitrogen supply, as nitrogen is a constitutive part of proteins, thereby providing stability to cells. A combination of different nano-N with PU in mustard has been reported to increase LAI and SPAD by 50%, at par with the control (Kumar and Chaitanya, 2022). Salinity stress at 120 mM NaCl drastically influenced plant growth parameters, physiological parameters, nutrient contents, antioxidant potential, as well as overall yield and yield-contributing attributes of maize plants. Apart from transpiration rate (TR) and intrinsic water use efficiency (WUE), foliar application of ZnO-NPs mitigated the adverse effects of salinity on LGR, PGR, etc., giving significant improvements in all studied parameters (Seleiman et al., 2023). Foliar application of an iron source has also been reported to improve SPAD values and chlorophyll content, along with preserving the stability of plant membranes in groundnut cultivars under limited availability of Fe, thus indicating the effectiveness and utility of foliar-applied micronutrients (Mann et al., 2017). All these findings align with our results, showing that the application of chelate-nitrogen (NPK_1_ and NPK_2_) and micronutrients enhances photosystem traits such as CCI, Pn, E, and WUE by balancing ionic and osmotic conditions, improves stomatal conductance, and maintains optimal gas exchange (**Figure 6**; **Table 2**) (Ahmed et al., 2024).

### 4.4 Enhancement of antioxidant defense system using nano-chelating fertilizers under salinity stress

Over-accumulation of NaCl in plant cells results in an oxidative stress that generates ROS, including O_2_^•−^, ^1^O_2_, and H_2_O_2_ (Mignolet-Spruyt et al., 2016; Wang et al., 2018). ROS can be detrimental damage to cellular components (lipids, proteins, and nucleic acids) of chloroplasts and mitochondria, resulting from a disrupted electron transport system (Faizan et al., 2024). To address this issue, antioxidant enzymes (SOD, CAT, APX, and GR) that modulate the harmful effects of ROS by the detoxification process (Sharma et al., 2012). The findings of the current experiment indicated that SOD and CAT under salt stress were significantly increased when foliar nano-chelating fertilizers (chelate-NPK_1_, NPK_2_, and chelate-micronutrient-complex) were applied. This increase was compared to the saline control, varying from slight to higher levels (**Table 3**). As suggested by the results of our study, controlled-release of NPK_1_, NPK_2_ fertilizers and nanonutrients (chelate-micronutrient-complex) enhanced enzymatic activities under stress conditions (Mehmood et al., 2021; Rady et al., 2023; Nazarideljou et al., 2024). It was also reported that wheat and oat plants showed marked increases in SOD and CAT activities when supplied with nano-chelating fertilizers under abiotic stress conditions (Rezaei and A, 2014; Rady et al., 2023). Likewise, studies confirm that MDA, a lipid peroxidation marker reflecting less oxidative damage from stresses, can be decreased by chelate-NPK and micronutrient-complex (Mazhar et al., 2023; Shao et al., 2023; Nazarideljou et al., 2024; Ding et al., 2025).The positive effects of Nano-NPK and micronutrient treatment are linked to increased stress tolerance, achieved through the regulation of antioxidants and a reduction in oxidative stress markers like MDA (57.85%) (Ding et al., 2025). Chelate-NPK_2_ and micronutrient-complex showed a slightly higher increase in the activity of antioxidant enzymes (SOD and CAT), alleviating oxidative damage (reduced MDA levels) and boosting resistance to salt stress of wheat and oat plants (**Table 3**).

Proline is another amino acid that accumulates during stress conditions in plants, acting as an osmoprotectant and stabilizing cytosolic pH, proteins and cell membranes while maintaining the osmotic potential within cells subjected to salinity stress (Ashraf and Foolad, 2007). The combined application of NPK_1_, NPK_2_ fertilizers and nanonutrients (chelate-micronutrient-complex) significantly enhanced tolerance in stressed plants by inducing proline levels. This might be a result of carboxylate synthase overexpression (key enzyme involved in proline biosynthesis) that enhances the plants ability to tolerate salinity stress (Rai and Penna, 2013). Another reason can be improved sugar metabolism with nano-chelating fertilizers via enzyme activity in key metabolic pathways, especially glycolytic and pentose phosphate pathways (Yang et al., 2022). All this helps to increase the total soluble sugars for osmotic adjustment when the plant is in stress conditions. Additionally, osmolyte regulation is improved, with proline assisting in this process in wheat and oat plants (Ding et al., 2025). Foliar nano-formulated NPK combined with Zn was studied to enhance sweet pepper (*Capsicum annuum* L., cv. ‘Dora’) nutritional quality (Koutelidakis et al., 2026). The optimal application of nano-NPK with 3 g/L Zn increased total phenolics, flavonoids, and vitamin C, thereby enhancing antioxidant activity, whereas 5 g/L Zn demonstrated dose-dependent metabolic modulation that led to physiological stress, indicated by higher proline levels. This suggests that nano-micronutrients help to enhance fruit quality and the avoidance of stress (Koutelidakis et al., 2026). However, the proline activities in both NPK and micronutrients (as well as chelate-micronutrient-complex) treatments exhibited increase, the cumulative results showed that NPK_1_, NPK_2_ nano-chelating and micronutrient-complex changed biochemical pathways and increased proline levels, particularly in the case of NPK_2_ and micronutrients complex slightly higher increase, compered other treatments. Such modulation enriches the plant’s salt stress tolerance, which has a significant positive influence on growth and productivity.

### 4.5 Improvement of yield parameters using nano-chelating fertilizers under salinity stress

Current research has shown that the use of nanochelated fertilizers, such as NPK_1_ (12:12:36), a balanced NPK_2_ (20:20:20), and a comprehensive micronutrient chelate complex (containing Fe-7%, Zn-1.5%, Mg-1.5%, Br-0.5%, Cu-0.5%, and Mo-0.5%), applied through the foliar method, significantly improved several wheat and oat yield traits under salinity stress. This finding suggests that balanced nutrient management is an important factor in increasing crop productivity (**Figure 7**). Because balanced application of nanochelated fertilizers allows better nutrient absorption and enhanced availability of fundamental elements for cellular development and growth. The current study found that using nanochelated NPK_1_ and NPK_2_ fertilizers with a comprehensive micronutrient chelate complex improved yield, which determines wheat and oat productivity under salinity stress conditions (Singh et al., 2024). Enhanced wheat and oat plant development and growth may have contributed to higher grain dry biomass per plant, which may be correlated with improved photosynthetic efficiency and greater nutritional availability (Salim and Raza, 2020; Zhang et al., 2025b). The use of nanochelated fertilizers boosted photosynthesis and overall plant development, which helps in increased tiller production through the promotion of branching and tillering (Scholar et al., 2023). Overall, the TKW was improved due to the balanced nutrient supply that came from using comprehensive micronutrient applications with nanochelated NPK_1,_ NPK_2_ and micronutrient fertilizers (Rezaei and A, 2014; Wu et al., 2018; Zhang et al., 2022; Mazhar et al., 2023; Shao et al., 2023). All of this occurred because the grains could produce higher amounts of proteins and starches, leading to an increased overall yield of wheat and oat plants (Shewry, 2024). Because the nanochelated fertilizer caused synergistic responses that impacted nutrient uptake, enzyme activity, and cellular metabolism, the end result showed that each plant boosted its overall grain output under saline conditions.

## 5 Conclusion

Overall, the use of proper and balanced nanochelated fertilizers has shown positive effects on wheat and oat growth, yield, and quality by modulating enzyme activity. Applying combined nanochelated fertilizers led to better growth traits, improved enzyme activity, and increased antioxidant capacity in these crops, while also reducing lipid peroxidation under salinity stress, compared to using nanochelated fertilizers alone. Effective nutrient management for wheat and oat cultivation is essential for sustainability, environmental protection, soil health, climate resilience, and food security. Future research should conduct field trials to confirm these findings and offer a more detailed comparison with traditional fertilizers at the molecular level, focusing on nutrient response mechanisms. Additionally, the development of improved nanochelated fertilizer management practices is key to establishing sustainable wheat and oat farming systems.

## Data Availability

The original contributions presented in the study are included in the article/supplementary material. Further inquiries can be directed to the corresponding authors.
